# Comparison of Tumor Seeding and Recurrence Rate After Laparoscopic vs. Open Nephroureterectomy for Upper Urinary Tract Transitional Cell Carcinoma

**DOI:** 10.3389/fsurg.2021.769527

**Published:** 2021-12-23

**Authors:** Simone Morselli, Ferdinando Daniele Vitelli, Giorgio Verrini, Arcangelo Sebastianelli, Riccardo Campi, Andrea Liaci, Pietro Spatafora, Paolo Barzaghi, Giovanni Ferrari, Mauro Gacci, Sergio Serni, Maurizio Brausi

**Affiliations:** ^1^Department of Experimental and Clinical Medicine, University of Florence, Florence, Italy; ^2^Unit of Urological Minimally Invasive Robotic Surgery and Renal Transplantation, Careggi Hospital, University of Florence, Florence, Italy; ^3^Department of Urology, Cure Hesperia Hospital, Modena, Italy; ^4^Department of Urology, AUSL Modena, Modena, Italy

**Keywords:** UTUC, nephroureretectomy, recurrence rate (RR), seeding, laparoscopy

## Abstract

**Introduction:** Laparoscopic surgery for Upper Urinary Tract Urothelial Cell Carcinoma (UTUC) is still debated for its possible seeding risk and thus consequent oncological recurrences, especially for atypical ones. The aim of the study is to compare recurrence and survival after Laparoscopic vs. Open Radical Nephroureterectomy (RNU) for Upper Urinary Tract Urothelial Cancer (UTUC).

**Method:** A retrospective evaluation of UTUC consecutive surgeries from 2008 to 2019 was conducted, including pT ≥ 2, High Grade UTUC who underwent RNU with bladder cuff excision without concomitant lymphadenectomy in three urological tertiary centers. Statistical analyses compared recurrence and cancer specific survival, based on surgical approach, while logistic multivariate analyses and Kaplan Meyer survival curve analyzed possible risk factors for recurrence and survival.

**Results:** One hundred seven cases of RNU, 47 (43.9%) laparoscopic and 60 (56.1%) open, were included in this report. Preoperative characteristics were comparable between groups. However, tumor stage was higher in the Open arm [T3–T4 in 44 (73.3%) vs. 20 (43.4%) in Laparoscopic]. Mean follow-up was 91.6 months in laparoscopy RNU vs. 93.5 months in open RNU. Recurrence rate (RR) was comparable between groups (*p* = 0.594), and so was the site, although 3 (6.3%) peritoneal recurrences were found only in laparoscopic group (*p* = 0.057). At multivariate logistic regression, tumor stage and surgical approach were independent predictors of recurrence (*p* < 0.05), while only tumor stage was predictor of cancer specific death (*p* = 0.029).

**Conclusion:** Surgical approach has no impact on recurrence site, overall survival, and RR. Still, according to our data peritoneal carcinomatosis was present only in laparoscopic arm, despite how it didn't reach statistical significance.

## Introduction

Upper Urinary Tract Urothelial Cell Carcinomas (UTUCs) are rare tumors, representing only 5% of all urothelial carcinomas, with a low annual incidence in Europe (1, 2). Despite their rarity, they are highly prone to recurrence and progression. As a consequence, radical nephroureterectomy (RNU) with bladder cuff excision is currently considered the gold standard for the curative management of high-risk UTUC. Kidney-sparing surgery, such as segmental ureteral resection or endoscopic ablation, could be considered in low-risk tumors for patients with solitary kidney and/or impaired renal function. RNU was historically performed via an open approach (ORNU). However, over time, the laparoscopic approach (LRNU) has been widespread as a minimally invasive approach, with less perioperative complications, faster recovery, and reduced costs (3). Nevertheless, some investigators reported the tumor dissemination and seeding under CO2 pneumoperitoneum during laparoscopic surgery with a consequent higher risk of bladder and/or local recurrence and port-site metastasis (4, 5). Therefore, the different effect of LRNU vs. ORNU on oncologic outcomes remains controversial (6–8). Thus, the aim of this study is to compare the rate and site of disease recurrence in ORNU and LRNU.

## Method

### Study Design and Patient Selection

A multicenter retrospective study involving three urologic tertiary centers was conducted, enrolling only patients with invasive UTUC who consecutively underwent RNU between 2008 and 2019. Inclusion criteria were as follows: stage ≥ pT2, high-grade (HG), no prior neoadjuvant chemotherapy neither concomitant lymph node dissection. All cases were performed by four well-experienced urological surgeons. The population was consequently divided according to surgical approach (ORNU vs. LRNU) without prior randomization.

### Preoperative Assessment

Gender, age, weight, BMI, American Society of Anesthesiologists (ASA) score, Charlson Comorbidity Index (CCI) smoking habit, comorbidities, and history of prior urothelial tumors were collected for each patient. Before surgery, all patients had undergone cystoscopy, urine cytology, and abdominal-pelvis computed tomography (CT) with urography phase plus thorax CT. Preoperative ureteroscopy was performed in doubt cases.

### Surgical Technique

All patients, regardless of surgical approach, underwent bladder cuff excision.

#### Laparoscopic RNU

Patient is placed in modified lateral position. First, a paraumbilical 12 mm trocar for optic is placed with Hasson technique, then pneumoperitoneum at 12 mmHg is induced and another two 10 mm trocar and one 5 mm trocar are placed. On right LRNU, a further 5 mm trocar below the xiphoid process to lift the liver with a grasp forceps might be needed. Standard transperitoneal adrenal sparing LRNU is usually performed without dissecting the ureter, which is then clipped with two hem-o-lok. Hence, kidney and the first ureteral tract are placed in an endobag. Therefore, trocars are removed and wounds closed. Patient is eventually placed in supine position and a low midline incision is made to access and isolate distal ureter until the Waldeyer's sheath. Lastly, an anterior cystotomy to complete bladder cuff excision is performed. Kidney, ureter, and bladder cuff are ultimately removed en-bloc.

#### Open RNU

A single midline incision or a double-incision approach (flank and low midline) were performed for the ORNUs.

### Pathologic Evaluation

All surgical specimens were processed in accordance with standard pathological procedures at each institution. Tumors were staged according to the American Joint Committee on Cancer TNM classification. Tumor grade was reported conforming to 2004/2016 WHO classification (9, 10).

### Follow-Up

No post-operative dose of intravesical chemotherapy was administered. Patients were followed according to EAU guidelines (11).

### Statistical Methods

Categorical and continuous variables were analyzed accordingly. Quantitative variables were expressed as mean ± standard deviation (SD) or median and interquartile range (IQR). Differences in recurrence rate (RR) between groups were compared using Fisher's exact test. Groups were then sub-divided according to pathological stage and compared consequently for recurrence, site of recurrence, and UTUC specific mortality. Binary logistic regression was performed to point out possible independent predictors of recurrence and death from UTUC. Results were reported as Odds Ratio (OR) and 95% Confidence Interval (CI). Kaplan-Meyer survival curve was realized to compare cancer specific survival according to surgical approach. Statistical significance was set at *p* < 0.05. Analyses were performed with Statistical Package for Social Sciences software, V.17.0 (SPSS Inc., Chicago, IL, USA).

## Results

### Clinical and Pathological Evaluation

After careful revision of the existing database, 107 patients were eligible and were, therefore, included in our study. Mean age was 72 years (*SD* = 7), while mean BMI was 25.8 kg/m2 (*SD* = 5.4). Forty-seven (43.9%) patients were female. Past or current smoking habit was present in 69 (64.4%) patients. UTUCs were located in the ureter in 42 cases (39.3%), in pelvis in 27 (25.2%), and were multifocal in 38 (35.5%). Hydronephrosis due to UTUC was present in 31 (29.0%) patients and urinary cytology was suspicious or positive in 81 (75.7%). Diagnostic preoperative Ureterorenoscopy (URS) was necessary in 44 (41.1%) patients. Past history of bladder cancer was present in 35 (32.7%) patients, of whom 5 (14.3%) had prior radical cystectomy (RC). Sixty (56.1%) patients were treated with ORNU, while 47 (43.9%) received LRNU. Patients' characteristics according to surgical approach are reassumed in Table 1. The two groups were comparable for age, BMI, gender, CCI, and ASA score (*p* > 0.05). Tumor characteristics were comparable for location (*p* = 0.662) and grade (HG in both groups), while stage was worst in open arm (*p* < 0.001) as ORNU had more T4 tumors: 12 (20.0%) vs. 3 (6.5%).

**Table 1:** Patients' characteristics according to surgical approach (Open vs. Laparoscopic) for Upper Urinary Tract Urothelial Cancer (UTUC) Nephroureterectomy.

		**Open RNU**	**Laparoscopic RNU**	***p***
		***n* = 60 (56.1%)**	***n* = 47** **(43.9%)**	
Age, years		73 (6)	70 (7)	0.029
BMI, kg/m^2^		25.5 (5.3)	25.9 (5.2)	0.643
Gender	Male	38 (63.3%)	22 (47.8%)	0.087
	Female	22 (36.7%)	25 (53.2%)	
ASA score		2 (2–3)	2 (2–3)	0.120
Charlson comorbidity index		3 (2–4)	3 (3–4)	0.851
Tumor location	Renal pelvis	12 (20.0%)	15 (34.1%)	0.662
	Ureter	25 (41.7%)	17 (36.2%)	
	Renal Pelvis plus Ureter	23 (38.3%)	15 (31.9%)	
Pathological stage	T2	16 (26.7%)	30 (56.6%)	<0.001
	T3	32 (53.3%)	15 (36.9%)	
	T4	12 (20.0%)	2 (6.5%)	
Grade	Low Grade	0 (0.0%)	0 (0.0%)	1.000
	High Grade	60 (100%)	46 (100%)	

*RNU, Radical Nephroureterectomy; BMI, body mass index; ASA, American Society of Anesthesiologists Statistic: Independent Sample t-test, Chi-Square test or Fisher's Exact test when appropriate. Variables are reported as Mean (Standard Deviation) or median (interquartile range) for categorical and n, (%) for nominal*.

### Site and Recurrence Rate

All patients underwent a complete follow-up. Mean follow-up was 89.3 months (*SD* = 34.4) in ORNU and 79.3 months (*SD* = 35.8) in LRNU, *p* = 0.148. Overall, 38 out of 107 patients experienced disease recurrence during follow-up: 20 (33.3%) in the ORNU group and 18 (38.3%) in the LRNU group. Among the 20 patients of ORNU arm, the sites of recurrence were lung and bone metastasis in 14 patients (70.0%) and lymph nodes in 6 (30.0%). During follow-up, bladder recurrence was detected in 16 patients (26.7%). No case of local recurrence was recorded in ORNU group. Eighteen patients who underwent LRNU recurred, 9 (50.0%) in lymph nodes, and 6 (33.0%) distally (bone or lung). There were three cases (17.0%) of peritoneal carcinomatosis and no cases of port site metastasis. RRs were similar in both arms (*p* = 0.594). When furtherly analyzed for recurrence site, no differences were observed between groups (*p* > 0.05), as showed in Table 2. According to Fisher's Exact Test, we found no difference in local RR (*p* = 0.057). However, at binary logistic regression, both tumor stage, and surgical approach were independent predictors of recurrence (*p* < 0.05), as reported in Table 3.

**Table 2:** Comparison of urothelial carcinoma recurrence incidence and Site according to surgical approach.

	**Type of approach**				
	**Open** **(*n* = 60)**		**Laparoscopic** **(*n* = 47)**		***p***
	***N***	**%**	***N***	**%**	
Recurrence (All)	20	33.3	18	38.3	0.594
Local	0	0.0	3	6.3	0.057
Nodal	6	10.0	9	19.1	0.208
Metastasis	14	33.3	6	12.8	0.638
Bladder	16	26.7	11	23.4	0.700

**Table 3:** Multivariate analysis for predictors of urothelial carcinoma recurrence in UTUC who underwent nephroureterectomy.

	**Odds**	**95% Confidence**	***p***
	**Ratio**	**Interval**	
Age	1.019	0.944–1.100	0.634
Body Mass Index	0.967	0.878–1.064	0.492
Surgical Approach	3.781	1.151–12.422	0.028
Tumor Stage	4.748	2.087–10.803	<0.001

### Death From UTUC

**Figure 1:**
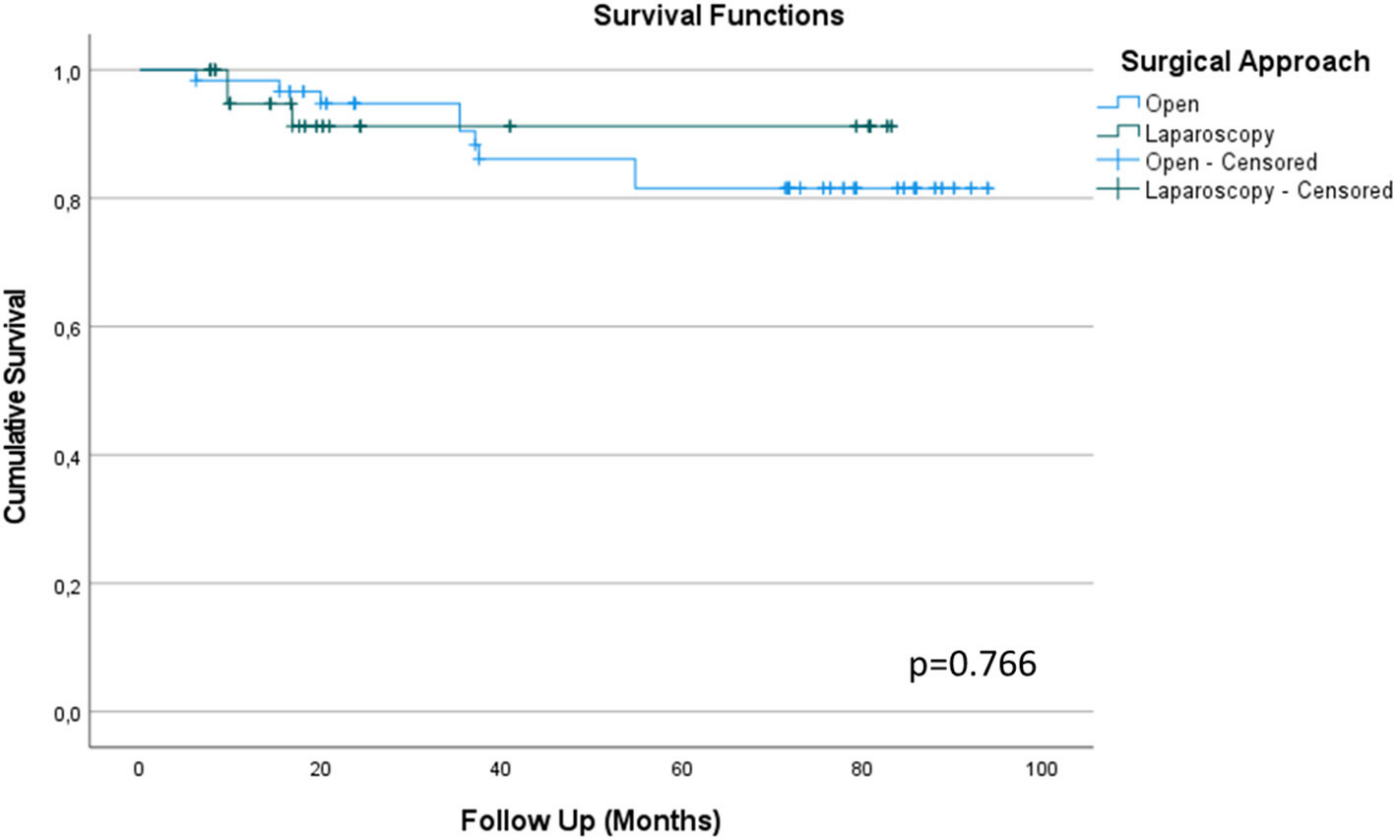
Kaplan-Meier survival comparison between Open and Laparoscopy surgical approach to Radical Nephroureterectomy for Upper Tract Urothelial Cancer (UTUC) based on cancer specific survival at long term follow-up.

During follow-up, 12 patients (11.2%) died from UTUC (excluding other death causes). In detail, 9 patients (75.0%) belonged to the ORNU group and 3 (25.0%) to the LRNU group, (*p* = 0.172). At age-adjusted binary logistic regression, tumor stage was the only independent predictor of death from UTUC, OR = 2.7 CI-95% = 1.1–6.6 (*p* = 0.029). In fact, T2 tumors were more represented in LRNU group, while T4 were more frequent in ORNU. In Figure 1, Kaplan-Meier survival comparison of UTUC according to surgical approach is displayed, thus showing comparable results.

### Oncological Outcomes According to Pathological Stage

A subsequent comparison by pathological stage between ORNU and LRNU groups was conducted. In detail, according to pathological stage, there were statistically significant differences between ORNU vs. LRNU, 6 (37.5%) vs. 2 (6.7%) *p* = 0.019 and 6 (18.8%) vs. 8 (53.3%) *p* = 0.009, respectively, only in intravesical recurrences in T2 and T3 patients. However, recurrences, from local to metastasis, were experienced only in LRNU arm in pT2, i.e., 24.0 vs. 0%, without reaching statistical significance (*p* = 0.055). In addition, local recurrences were reported even in pT3 only in LRNU group, 22.3 vs. 0%, but also in this case in a non-significant way (*p* = 0.065). All data are reported in Supplementary Table 1.

## Discussion

Open Radical Nephroureterectomy (ORNU) with bladder cuff excision is the standard approach for high-risk UTUC regardless of tumor location despite how other techniques might be viable alternatives in selected cases (11). Nevertheless, the advantages of laparoscopic procedures in terms of perioperative course and patient recovery have made this technique very appealing through the years (12). On the other hand, concerns about the oncologic safety of LNU have been raised during the last two decades because of the incidence of port-site metastases and the supposed role of high-pressure environment of pneumoperitoneum in tumor-cell seeding (13). However, it is known that several precautions and the respect of oncological principles of open surgery may lower the risk of tumor spillage (5). In this study, only high-risk UTUCs (stage ≥ pT2 and HG disease) without nodal invasion or distant metastasis at diagnosis were included. None of our patients received lymphadenectomy, which may have an impact on survival, as seen in some studies (14, 15).

### Recurrence

According to our results, we registered similar tumor RR between ORNU and LRNU arms. Particularly, at binary logistic regression, surgical approach was deemed related to higher RR. However, tumor stage was almost unbalanced between groups, and it was also the strongest predictor of recurrence. Therefore, the statistically significant relationship of the approach as predictor of recurrence should be addressed to that. Interestingly, we did not report any case of port-site metastases. These results perfectly fit in current literature as in the only published prospective randomized study, the authors proved the oncological safety and the similar results of ORNU and LRNU for patients with HG organ-confined disease (pT < 3) (7). Notably, when ORNU and LRNU were stratified and compared according to pathological stage, only LRNU had recurrence in pT2 tumors despite not reaching statistical significance (*p* = 0.055), which is in disagreement with currently reported literature. Indeed, according to guidelines, for extensive or invasive tumors (pT3/T4 and/or N+/M+), laparoscopic approach is not recommended because of its poorer oncological outcome when compared with open surgery. Nevertheless, in our study, we did not find a relevant difference between surgical approach and oncological results for higher stages, possibly due to the fact that were small unbalanced groups at sub-analysis (6, 7, 11).

### Site of Recurrence

Currently, atypical sites of recurrence and port-site metastasis have been reported in literature after LRNU for UTUC (16). In our population, a similar proportion of retroperitoneal lymph node metastasis was recorded in the two arms, 10.0 vs. 19.1%, respectively, *p* = 0.208. There are contrasting data regarding the regional node recurrence after nephroureterectomy. Particularly, two large multicenter studies had registered a higher but not significant incidence in the open arm (17, 18). On the contrary, our results are supported by the homogeneity of the two subgroups in terms of preoperative N staging, that was negative for all the patients, which did not allow the performance of lymph node dissection in any case (14). Interestingly, we found two cases (4.3%) of peritoneal carcinosis which occurred at month 3 and 4 after LRNU with a consequent negative impact on their outcomes. On the contrary, open arm had no cases of peritoneal recurrences, thus, this difference was almost statistically significant at Fisher's exact test (*p* = 0.057). Moreover, at sub-analysis, only in pT2 LRNU local recurrence was present. This is noticeable while considering the higher rate of higher pathological stage UTUC in ORNU compared to LRNU arm (*p* < 0.001). Nevertheless, cases of peritoneal cancer dissemination have been reported in literature, always for high-grade invasive tumors regardless of the surgical approach (5). Indeed, Hemal et al. preferred a retroperitoneal approach in LRNU to prevent peritoneal contamination of tumor cells (19). Also, Carrion et al. reported cases of atypical recurrence following LRNU, both peritoneal and in trocar site, relating them to laparoscopic approach (20). Indeed, all patients included in the laparoscopic group of our study received a transperitoneal access, according to surgeon's preference and expertise.

### Bladder Recurrence

Bladder cuff excision was performed through an open approach in all cases considered in this study, with the patient in supine position. Indeed, bladder tumor recurrence was similar in both groups, irrespective to the chosen approach (26.7 vs. 23.4% in open vs. laparoscopic group, respectively, *p* = 0.700). Only at sub-analysis was a difference was present, particularly in pT2 higher in ORNU (37.5 vs. 6.7%, *p* = 0.015) and in pT3 higher in LRNU (18.8 vs. 53.3% *p* = 0.009), but differences at sub-analysis appear irrelevant due to the sample dimension of each sub-group. Nevertheless, our results are slightly more encouraging of those reported in a recent systematic review and meta-analysis of a bladder RR of about 30% after RNU (21). Moreover, no adjuvant chemotherapy with instillation of mitomycin C, which can furtherly lower the intravesical RR, was administered in our cohort (22).

### Possible Mechanism of Peritoneal Tumor Seeding During Laparoscopy

Laparoscopy oncological safety has been questioned many times. Indeed, evidences suggest that, while globally preserving immune system function, laparoscopy may reduce it locally, thus severely affecting macrophage function (23, 24). Nevertheless, knowledge about pathogenetic mechanisms behind tumor seeding and port-site metastasis are limited. *In vitro* and *in vivo* studies investigated the role of laparoscopy and its chosen gas (CO2 vs. Xenon or Helium) while some nuances may lower seeding risks (25). In particular, conditioning or humidification of CO2 lowered peritoneal tumor implantation rate in a mice model (26). Although there are no randomized clinical trials in urologic surgery and, in particular, in UTUC, a similar study conducted in 2018 on gynecologic surgery evaluating the oncological risk intrinsically related to laparoscopy might change the clinical practice in gynecologic malignancies. Thus, such a trial is advocated even in UTUC (27). Currently, tumor cells seeding after minimally invasive surgery seems under reported in literature, as urothelial carcinoma is one of the most malignant urological tumors, with a high RR. In fact, most of the published data on tumor seeding and port site metastases in urological literature are related to it (13). Actually, a retrospective review of 338 patients who underwent open (*N* = 120) or robotic (*N* = 253) RC from 2000 to 2014 looked for the recurrence patterns and anatomical location of metastases within 2 years of surgery. After a median follow-up time of 30 months, local and distant RR were similar in both arms. However, further analysis on distant recurrences patterns revealed that extra pelvic lymph node locations were 23 vs. 15% and peritoneal carcinomatosis were 21 vs. 8% in robotic vs. open RC, respectively. Thus, both of them were more frequent in robotic RC (28). Another critical analysis of early recurrences after laparoscopic RC in a large cohort of 331 patients with favorable pathology (pT2N0R0) was conducted by Albisinni et al. within 24 months with 27 (8.7%) experienced recurrences. Among them, CIS and a shorter recurrence free survival were independent predictors for cancer specific death. Moreover, some of them with T0–T1 tumor developed disseminated bone metastasis in <12 months and, in some patients, atypical metastatic sites were observed (29). A hypothetic cause could be the continuous insufflation-desufflation and leakage of gas around the ports with consequent aspiration of tumor cells in a chimney that can promote tumor seeding. This was also showed by Mynbaev et al. *in svitro* (30). Indeed, the etiological factors of tumor seeding can also be related to tumor, wound, and surgical technique. When we analyze proper tumor risk factors for seeding, they can be its grade and stage, concomitant CIS or DNA (tumor aneuploidy). Undeniably, HG urothelial carcinoma represents the majority of tumor seeding and port-site metastases in urological procedures (13). Wound-related seeding factors might be sought in pneumoperitoneum. Its physiopathological mechanism is not completely defined, but it has been suggested that a reduction of peritoneal PH can lead to an increase in vascular permeability and, thus, to an alteration of adhesion molecules, potentially promoting tumor spreading and seeding (5, 24). Moreover, CO2 insufflation may determine macrophage function alteration and, therefore, immune-depression that allows cancer cells to escape surveillance (24). Surgical technique-related tumor seeding factors might be identified in three delicate surgical steps, which may be exacerbated in robotic or laparoscopy that may determine a tumor cells spillage. First, in lymph node dissection because nodes should be removed intact from chains as their rupture and damage results in cancer cells dissemination, especially if lymphovascular invasion is present (mostly pT3–T4). However, in our study, lymph node dissection was not performed. Second, a correctly performed RNU because kidney and ureters should be removed intact while looking for negative margins. Ureters should be clipped before excision to avoid cancer cells spillage with urine. Third, during the specimen removal, kidney and ureters with eventually lymph node chains must be put in bags as soon as they are free and then promptly retrieved. Therefore, immunodepression determined by pneumoperitoneum and imperfect surgery can together explain peritoneal carcinomatosis cases.

## Limitations

Our study limitations are its retrospective design and absence of a standardized surgical approach. Indeed, lack of randomization gave unbalanced groups. In fact, the number of LRNU was inferior while in the ORNU group there were higher stage patients (T3–T4). Hence, affecting results, especially at sub-analysis for pathological stage. Thus, these factors partly impact on both RR and survival. Certainly, another limitation is related to the absence of a lymphadenectomy in our patients, which may positively influence patients' survival. Besides all these weaknesses, this study provides evidences that LRNU has similar outcomes than ORNU, but it should be taken in account that peritoneal carcinomatosis was observed only in LRNU (6.3 vs. 0%) which yielded lower stage UTUC.

## Conclusions

Based on our results, ORNU and LRNU have globally similar RR and outcomes at long term follow-up. Nevertheless, peritoneal carcinosis occurred exclusively with laparoscopy and even in pT2 patients. Therefore, suggesting that further well-designed multi-institutional randomized controlled trials are mandatory to clarify the influence of laparoscopy and robotic on atypical recurrence site in UTUC and their subsequent oncological impact on survival.

## Data Availability Statement

The original contributions presented in the study are included in the article/Supplementary Material, further inquiries can be directed to the corresponding author/s.

## Ethics Statement

Ethical review and approval was not required for the study on human participants in accordance with the local legislation and institutional requirements. The patients/participants provided their written informed consent to participate in this study.

## Author Contributions

SM, FV, GV, and AS: study design. AL, PS, and PB: data collection. SM, RC, GF, and MG: manuscript writing. SM, RC, AS, MG, and PB: critical revision of the manuscript. SS and MB: supervision. All authors contributed to the article and approved the submitted version.

## Author Disclaimer

All claims expressed in this article are solely those of the authors and do not necessarily represent those of their affiliated organizations, or those of the publisher, the editors, and the reviewers. Any product that may be evaluated in this article or claim that may be made by its manufacturer is not guaranteed or endorsed by the publisher.

## Conflict of Interest

The authors declare that the research was conducted in the absence of any commercial or financial relationships that could be construed as a potential conflict of interest.

## Publisher's Note

All claims expressed in this article are solely those of the authors and do not necessarily represent those of their affiliated organizations, or those of the publisher, the editors and the reviewers. Any product that may be evaluated in this article, or claim that may be made by its manufacturer, is not guaranteed or endorsed by the publisher.

## References

[1] Siegel RL Miller KD Jemal A. Cancer statistics, 2017. CA Cancer J Clin. (2017) 67:7–30. 10.3322/caac.21387 28055103

[2] Munoz JJ Ellison LM. Upper tract urothelial neoplasms: incidence and survival during the last 2 decades. J Urol. (2000) 164:1523–5. 10.1016/S0022-5347(05)67019-X 11025695

[3] Hanna N Sun M Trinh QD Hansen J Bianchi M Montorsi F. Propensity-score-matched comparison of perioperative outcomes between open and laparoscopic nephroureterectomy: a national series. Eur Urol. (2012) 61:715–21. 10.1016/j.eururo.2011.12.026 22209172

[4] Ni S Tao W Chen Q Liu L Jiang H Hu H. Laparoscopic versus open nephroureterectomy for the treatment of upper urinary tract urothelial carcinoma: a systematic review and cumulative analysis of comparative studies. Eur Urol. (2012) 61:1142–53. 10.1016/j.eururo.2012.02.019 22349569

[5] Rouprêt M Smyth G Irani J Guy L Davin JL Saint F. Oncological risk of laparoscopic surgery in urothelial carcinomas. World J Urol. (2009) 27:81–8. 10.1007/s00345-008-0349-x 19020880

[6] Peyronnet B Seisen T Dominguez-Escrig JL Bruins HM Yuan CY Lam T. Oncological outcomes of laparoscopic nephroureterectomy versus open radical nephroureterectomy for upper tract urothelial carcinoma: an European association of urology guidelines systematic review. Eur Urol Focus. (2019) 5:205–23. 10.1016/j.euf.2017.10.003 29154042

[7] Simone G Papalia R Guaglianone S Ferriero M Leonardo C Forastiere E. Laparoscopic versus open nephroureterectomy: perioperative and oncologic outcomes from a randomised prospective study. Eur Urol. (2009) 56:520–6. 10.1016/j.eururo.2009.06.013 19560259

[8] Walton TJ Novara G Matsumoto K Kassouf W Fritsche HM Artibani W. Oncological outcomes after laparoscopic and open radical nephroureterectomy: results from an international cohort. BJU Int. (2011) 108:406–12. 10.1111/j.1464-410X.2010.09826.x 21078048

[9] Humphrey PA Moch H Cubilla AL Ulbright TM Reuter VE. The 2016 WHO classification of tumours of the urinary system and male genital organs—Part B: prostate and bladder tumours. Eur Urol. (2016) 70:106–19. 10.1016/j.eururo.2016.02.028 26996659

[10] Montironi R Lopez-Beltran A. The 2004 WHO classification of bladder tumors: a summary and commentary. Int J Surg Pathol. (2005) 13:143–53. 10.1177/106689690501300203 15864376

[11] Rouprêt M Babjuk M Burger M Capoun O Cohen D Compérat EM. European association of urology guidelines on upper urinary tract urothelial carcinoma: 2020 update. Eur Urol. (2020) 79:62–79. 10.1016/j.eururo.2020.05.042 32593530

[12] Lee H Kim HJ Lee SE Hong SK Byun SS. Comparison of oncological and perioperative outcomes of open, laparoscopic, and robotic nephroureterectomy approaches in patients with non-metastatic upper-tract urothelial carcinoma. PLoS ONE. (2019) 14:e0210401. 10.1371/journal.pone.0210401 30620766 PMC6324816

[13] Micali S Celia A Bove P De Stefani S Sighinolfi MC Kavoussi LR. Tumor seeding in urological laparoscopy: an international survey. J Urol. (2004) 171:2151–4. 10.1097/01.ju.0000124929.05706.6b 15126775

[14] Roscigno M Brausi M Heidenreich A Lotan Y Margulis V Shariat SF. Lymphadenectomy at the time of nephroureterectomy for upper tract urothelial cancer. Eur Urol. (2011) 60:776–83. 10.1016/j.eururo.2011.07.009 21798659

[15] Brausi MA Gavioli M De Luca G Verrini G Peracchia G Simonini G. Retroperitoneal lymph node dissection (RPLD) in conjunction with nephroureterectomy in the treatment of infiltrative transitional cell carcinoma (TCC) of the upper urinary tract: impact on survival. Eur Urol. (2007) 52:1414–8. 10.1016/j.eururo.2007.04.070 17507148

[16] Ong AM Bhayani SB Pavlovich CP. Trocar site recurrence after laparoscopic nephroureterectomy. J Urol. (2003) 170:1301. 10.1097/01.ju.0000084660.73614.da 14501747

[17] Ariane MM Colin P Ouzzane A Pignot G Audouin M Cornu JN. Assessment of oncologic control obtained after open versus laparoscopic nephroureterectomy for upper urinary tract urothelial carcinomas (UUT-UCs): results from a large French multicenter collaborative study. Ann Surg Oncol. (2012) 19:301–8. 10.1245/s10434-011-1841-x 21691878

[18] Capitanio U Shariat SF Isbarn H Weizer A Remzi M Roscigno M. Comparison of oncologic outcomes for open and laparoscopic nephroureterectomy: a multi-institutional analysis of 1249 cases. Eur Urol. (2009) 56:1–9. 10.1016/j.eururo.2009.03.072 19361911

[19] Hemal AK Kumar A Gupta NP Seth A. Retroperitoneal nephroureterectomy with excision of cuff of the bladder for upper urinary tract transitional cell carcinoma: comparison of laparoscopic and open surgery with long-term follow-up. World J Urol. (2008) 26:381–6. 10.1007/s00345-008-0265-0 18431579

[20] Carrion A Huguet J García-Cruz E Izquierdo L Mateu L Musquera M. Intraoperative prognostic factors and atypical patterns of recurrence in patients with upper urinary tract urothelial carcinoma treated with laparoscopic radical nephroureterectomy. Scand J Urol. (2016) 50:305–12. 10.3109/21681805.2016.1144219 26926709

[21] Seisen T Granger B Colin P Léon P Utard G Renard-Penna R. A systematic review and meta-analysis of clinicopathologic factors linked to intravesical recurrence after radical nephroureterectomy to treat upper tract urothelial carcinoma. Eur Urol. (2015) 67:1122–33. 10.1016/j.eururo.2014.11.035 25488681

[22] Fang D Li XS Xiong GY Yao L He ZS Zhou LQ. Prophylactic intravesical chemotherapy to prevent bladder tumors after nephroureterectomy for primary upper urinary tract urothelial carcinomas: a systematic review and meta-analysis. Urol Int. (2013) 91:291–6. 10.1159/000350508 23948770

[23] Ng CS Whelan RL Lacy AM Yim AP. Is minimal access surgery for cancer associated with immunologic benefits? World J Surg. (2005). 29:975–81. 10.1007/s00268-005-0029-6 15981046

[24] Ost MC Patel KP Rastinehad AR Chu PY Anderson AE Smith AD. Pneumoperitoneum with carbon dioxide inhibits macrophage tumor necrosis factor-alpha secretion: source of transitional-cell carcinoma port-site metastasis, with prophylactic irrigation strategies to decrease laparoscopic oncologic risks. J Endourol. (2008) 22:105–12. 10.1089/end.2007.9858 18315481

[25] Jacobi CA Bonjer HJ Puttick MI O'Sullivan R Lee SW Schwalbach P. Oncologic implications of laparoscopic and open surgery. Surg Endosc. (2002) 16:441–5. 10.1007/s00464-001-8112-z 11928024

[26] Binda MM Corona R Amant F Koninckx PR. Conditioning of the abdominal cavity reduces tumor implantation in a laparoscopic mouse model. Surg Today. (2014) 44:1328–35. 10.1007/s00595-014-0832-5 24452508 PMC4055846

[27] Ramirez PT Frumovitz M Pareja R Lopez A Vieira M Ribeiro R. Minimally invasive versus abdominal radical hysterectomy for cervical cancer. N Engl J Med. (2018) 379:1895–904. 10.1056/NEJMoa1806395 30380365

[28] Nguyen DP Al Hussein Al Awamlh B Wu X O'Malley P Inoyatov IM Ayangbesan A. Recurrence patterns after open and robot-assisted radical cystectomy for bladder cancer. Eur Urol. (2015) 68:399–405. 10.1016/j.eururo.2015.02.003 25709026 PMC4727829

[29] Albisinni S Fossion L Oderda M Aboumarzouk OM Aoun F Tokas T. Critical analysis of early recurrence after laparoscopic radical cystectomy in a large cohort by the ESUT. J Urol. (2016) 195:1710–7. 10.1016/j.juro.2016.01.008 26796414

[30] Mynbaev OA Baimaganbetov AK Eliseeva MY. Is there any adhesiogenic impact of CO2-pneumoperitoneum: pro and contra findings. Int J Surg. (2015) 23:115–7. 10.1016/j.ijsu.2015.09.050 26403070

