# Supplementary material for: Comparison of Tumor Seeding and Recurrence Rate After Laparoscopic vs. Open Nephroureterectomy for Upper Urinary Tract Transitional Cell Carcinoma
Source: Front Surg. 2021 Dec 23;8:769527. doi: 10.3389/fsurg.2021.769527 (PMC8732869; doi:10.3389/fsurg.2021.769527)
Supplement: Supplementary file 1 [file Table_1.DOCX]

Supplementary Table 1 – Oncological outcomes according to Pathological Stage at radical nephroureterectomy

| Pathological Stage | | Type of Approach | | | | |
| --- | --- | --- | --- | --- | --- | --- |
|  |  | Open | | Laparoscopic | | p |
|  |  | N=16 | % | N=30 | % |  |
| T2 (n=46) | Recurrences | 0 | 0% | 6 | 24.0% | 0.055 |
|  | Local | 0 | 0% | 2 | 33.3% | / |
|  | Nodal | 0 | 0% | 4 | 66.7% | / |
|  | Metastasis | 0 | 0% | 0 | 0% | / |
|  | Bladder | 6 | 37.5% | 2 | 6.7% | **0.015** |
|  | Death by UTUC | 0 | 0% | 0 | 0% | / |
|  | | N=32 | % | N=15 | % | p |
| T3 (n=47) | Recurrences | 14 | 43.8% | 10 | 66.7% | 0.180 |
|  | Local | 0 | 0% | 1 | 10.0% | 0.065 |
|  | Nodal | 3 | 21.4% | 4 | 40.0% | 0.108 |
|  | Metastasis | 11 | 78.6% | 5 | 50.0% | 0.242 |
|  | Bladder | 6 | 18.8% | 8 | 53.3% | **0.009** |
|  | Death by UTUC | 8 | 25.0% | 3 | 20.0% | 0.794 |
|  | | N=12 | % | N=2 | % | p |
| T4 (n=14) | Recurrences | 6 | 50% | 2 | 100% | 0.186 |
|  | Local | 0 | 0.0% | 0 | 0% | / |
|  | Nodal | 3 | 50% | 1 | 50% | 1 |
|  | Metastasis | 3 | 50% | 1 | 50% | 1 |
|  | Bladder | 4 | 33.3% | 0 | 0% | 0.334 |
|  | Death by UTUC | 1 | 8.3% | 0 | 0% | 0.672 |
